# Clinician Compliance With DVLA Guidelines in a Female Acute Inpatient Unit: Evaluating Documentation and Advice on Driving Status

**DOI:** 10.1192/bjo.2025.10680

**Published:** 2025-06-20

**Authors:** Alka Adhikari, Noemi Stockdale

**Affiliations:** North London NHS Foundation Trust, London, United Kingdom

## Abstract

**Aims:** The aim of this study was to assess clinician compliance with DVLA guidelines regarding driving status and advice for patients in an acute female inpatient unit. Specifically, the study sought to identify the documentation of driving status in patient records, the provision of DVLA-compliant advice, and the effectiveness of implemented recommendations.

**Methods:** This retrospective study reviewed electronic records of 20 patients admitted to an acute inpatient female ward. Data collection focused on whether driving status was documented and if DVLA-compliant advice was provided. Following the initial data collection, interventions were introduced, including visual prompts and modifications to the discharge summary template. The records of an additional 20 patients, admitted after these interventions, were subsequently reviewed. Data analysis was conducted using Microsoft Excel.

**Results:** Before implementing the recommendations, 20% of patients had their driving status recorded, and 15% received relevant advice. None of the discharge summaries included information about driving status. After the recommendations, the proportion of patients with recorded driving status increased to 25%, with 20% receiving relevant advice. Additionally, 25% of discharge summaries included driving-related information. The most significant improvement was observed in the inclusion of driving status in discharge summaries, attributed to the modified template. However, overall documentation and provision of advice remained suboptimal, indicating the need for further improvements.

**Conclusion:** The introduction of visual prompts and discharge summary template modifications led to modest improvements in the documentation and provision of DVLA-compliant driving advice. The findings suggest that additional interventions, such as enhanced data collection at initial patient reviews and clerking, could further improve compliance. Future efforts should focus on integrating these practices into routine clinical workflows to ensure comprehensive documentation and patient safety.

